# Screening tools used by paediatric healthcare providers to identify child maltreatment by parents or caregivers: a systematic review

**DOI:** 10.1136/bmjopen-2025-101721

**Published:** 2025-08-04

**Authors:** Josefine Ejnell Bjursell, Helena Wigert, Katarina Patriksson, Stefan Nilsson

**Affiliations:** 1 Division of Neonatology, Sahlgrenska University Hospital, Gothenburg, Sweden; 2 Institute of Health and Care Sciences, University of Gothenburg, Gothenburg, Sweden; 3 Department of Health Sciences, University West, Trollhättan, Sweden; 4 Centre for Person-Centred Care, University of Gothenburg, Gothenburg, Sweden; 5 Region Västra Götaland, Sahlgrenska University Hospital, Queen Silvia Children′s Hospital, Gothenburg, Sweden

**Keywords:** Child protection, PAEDIATRICS, Caregivers

## Abstract

**Abstract:**

**Objective:**

To evaluate the reliability and validity of screening tools designed to identify child maltreatment by parents or caregivers in paediatric healthcare settings, particularly for use in early childhood or neonatal care.

**Design:**

Systematic literature review.

**Data sources:**

The Cochrane Library, Embase, Cinahl and Ovid Medline were searched for studies published up to June 2025. Eligibility criteria for selecting studies: studies evaluating screening tools intended for use by caregivers or healthcare professionals to identify child maltreatment in paediatric healthcare settings. Included tools targeted children under 18 years of age.

**Data extraction and synthesis:**

Key characteristics of included tools were extracted, including type of maltreatment assessed, number of items, tool format and the age range of the child population. Data on reliability and validity were synthesised narratively due to heterogeneity in methods and outcomes.

**Quality appraisal:**

Risk of bias and the quality of Patient Reported Outcome Measure development were assessed using the Consensus-based Standards for the Selection of Health Measurement Instruments checklist, which also guided the grading of evidence strength.

**Results:**

In total, 1874 abstracts and 84 full-text articles were reviewed. 14 articles featuring 13 distinct screening tools were identified, most of which were used to detect physical abuse in emergency room settings. Only the Escape tool was evaluated in two studies, both of very good quality; the study evaluating the Pediatric Hurt-Insult-Threaten-Scream-Sex tool also demonstrated very good quality. The remaining studies varied in methodological quality and evidence strength. No tools were identified for children in neonatal care settings, and few were validated for children aged 0–3 years, none for neonatal care.

**Conclusion:**

This review highlights the limited number of validated tools suitable for identifying maltreatment in very young children and across different contexts, particularly neonatal care. Future research should address these critical gaps to better support the early identification of child abuse within diverse clinical settings.

**PROSPERO registration number:**

PROSPERO (2023), registration number CRD42023483966.

**STRENGTHS AND LIMITATIONS OF THIS STUDY**

- This review followed a comprehensive, pre-registered protocol in accordance with Preferred Reporting Items for Systematic Reviews and Meta-Analysis guidelines.
- Using Consensus-based Standards for the Selection of Health Measurement Instruments to assess the risk of bias and the quality of Patient-Reported Outcome Measures and Grading of Recommendation, Assessment, Development and Evaluation for evaluating the strength of the evidence

## Introduction

 Child maltreatment includes physical, sexual and emotional abuse and neglect, while exposure to intimate partner violence can also constitute a form of child abuse. Approximately 300 million children aged 2–4 years suffer maltreatment, but the study of statistics is difficult, as they vary depending on the country and methods used. The child is mostly maltreated by caregivers or others in authority, and the negative effects are immediate, with risk of lifelong consequences for physical and mental health, risk-taking behaviours and overall quality of life. Child maltreatment is often hidden, and only a small number of victims obtain support from healthcare professionals. Risk factors for being a victim of child maltreatment include being aged under 4 years or an adolescent, being unwanted or failing to fulfil the parents’ expectations, having special needs, crying persistently or having abnormal physical features and having an intellectual disability or neurological disorder. For the caregiver, the risk factors could be, for example, difficulty bonding with the newborn, not nurturing the child, having unrealistic expectations or lacking awareness of the child’s development and being maltreated themselves as a child. The use of substances (alcohol or drugs), also during pregnancy, having low self-esteem, poor impulse control, a mental or neurological disorder, being involved in criminal activity or experiencing financial difficulties could also be risk factors. There are also community risk factors, for example, social and cultural norms which could characterise the relationships within families or among intimate partners, friends and peers. Child maltreatment can be prevented by interventions, for example, supporting parents and caregivers, promoting better norms and values, education as well as creating and sustaining safe environments for children.^1 2^

All children have the right to grow up in a safe environment and society has a responsibility for children exposed to or at risk of child maltreatment. However, many children who are exposed are not reported to the social services and cannot obtain the protection they need. In Sweden, as in many other countries, professionals in, for example, schools, healthcare and dental care are obliged by law to report suspected child maltreatment to the social services.^3^ There may be various reasons for the underreporting of child maltreatment, such as difficulties discussing the suspicion with the parents, mainly due to practical problems such as limited time, lack of a suitable location and personal barriers, such as fear of an unjustified suspicion. Nurses experience concern about submitting an unsubstantiated report because of how it would affect the family. There are worries about upsetting the family if the maltreatment suspicions prove to be unfounded.^4 5^

In the Swedish healthcare context, particularly within nursing and care for young children, it is crucial to use evidence-based screening instruments that are both specific and sensitive in detecting suspected child maltreatment. Such tools should have a high number needed to treat, effectively identifying families genuinely in need of intervention, while maintaining a low number needed to harm to avoid falsely accusing innocent families. This precision is essential to foster trust between families and healthcare professionals, ensure that resources are appropriately allocated and prioritise the child’s safety without causing undue harm.

An updated systematic review is warranted to assess whether new studies have emerged that further evaluate the instruments identified by Chen *et al *^6^ or if any new screening tools have been developed—particularly those applicable to neonatal care settings. The present review serves as a foundation for the implementation of a tool designed to support specialist physicians and nurses in Swedish neonatal care in the identification of maltreatment.

## Methods

### Study design

The aim of this review is to update the search for studies on screening tools used in paediatric care to identify child maltreatment and to evaluate their validity and reliability. For this review, we focus on young children, specifically infants—including those receiving care in neonatal settings—within paediatric healthcare. However, our research spans children aged 0–18 years, aiming to update the review conducted by Chen *et al*.^6^ The rationale for extending the age range from infants and toddlers to the age of 18 years is that many instruments cover a broader span, reducing the risk of missing cases with shorter age ranges. We define child maltreatment as a broad spectrum of abuse, including physical, emotional and sexual abuse, as well as neglect. Our review takes a blended approach, considering various forms of harm. We aim to examine screening tools used to detect child maltreatment across these categories. Psychological and physical abuse often occur together and may later escalate into sexual abuse. We tend to search for tools typically used by nurses or doctors. The primary settings for these screenings are paediatric hospital wards and emergency rooms.

The review included tools designed for use by healthcare professionals such as paediatricians, emergency physicians, nurses and—in some cases—primary care providers. However, some studies did not clearly specify the level of training of the professionals involved, which we acknowledge as a limitation.

Paediatric care was defined broadly as healthcare services for individuals under 18 years of age, delivered in settings ranging from hospital-based (emergency departments, inpatient wards, paediatric intensive care unit) to outpatient clinics and, in some cases, community-based environments such as primary care or home visits.

This systematic review is part of a larger project aimed at identifying and synthesising evidence related to screening and addressing child maltreatment, as well as examining factors that influence the knowledge translation process.

The database selection was guided by the goal of capturing a comprehensive body of literature to thoroughly address the research question. The study is registered in PROSPERO (2023), registration number CRD42023483966. It is available at: https://www.crd.york.ac.uk/prospero/display_record.php?ID=CRD42023483966.^7^

### Search strategy

A clinical librarian performed the literature search in the Cochrane Library, Embase, Cinahl and Ovid Medline databases, for studies of screening tools used by caregivers and healthcare professionals in paediatric care to identify child abuse and neglect (CAN).

A systematic literature search was first conducted in November 2023 and subsequently updated in June 2025 to ensure the inclusion of the most recent studies. The original and updated search strategies are provided in online supplemental tables 1A–D and 2A–D, respectively.

#### Inclusion criteria

Studies published in English, including healthcare professionals, hospitals, clinics or wards, as well as parents and children aged 0–18 years. The screening tools identified must be used for child maltreatment detection in healthcare. The studies should also include validity and reliability testing of Patient Reported Outcome Measurements (PROM).

#### Exclusion criteria

Monographs, theses, books, editorials and systematic reviews were excluded.

The following keywords were used in the literature search: newborn, babies, baby, child, children, detect* infant, infants, instrument, maltreatment, measure*, mistreatment, neglect, neonat*, preterm, scale, screening tool*, CAN, toddler, adolescence, teenage, young people and abuse (online supplemental table 1A–D for the initial search and online supplemental table 2A–D for the updated search). Before the final analysis, a re-run of the searches took place. Duplicates were excluded and an EndNote file was uploaded to Rayyan, an online screening tool.^8^

### Search outcome

The title and abstract of studies (n: 1874) were blindly assessed for eligibility by two researchers using Rayyan (ie, each researcher’s decision on a study was hidden from the other researcher). The decision in Rayyan could be either to include, exclude or reconsider. Discrepancies in study selection were discussed between the two researchers. The result of full text articles (n: 84) was assessed by four blinded researchers. Discrepancies in study selection were discussed between the four researchers.

Data extraction was made by identifying different domains such as form of abuse, country, year, number of items in the form, tool, age, number of patients, sensitivity, specificity and area under the curve (AUC).

The Consensus-based Standards for the selection of health status Measurement Instruments (COSMIN) checklist^9^ was developed to evaluate the methodological properties of health-related patient reported outcomes (HR-PROs). The included studies were evaluated using COSMIN. The goal of a systematic review of PROMs is to recommend the most suitable PROM for the context of interest. The PROM needs to have been validated to at least some extent. One step in COSMIN is to grade the quality of evidence in order to rate the measurement property. For example, the reviewer will be more confident that a PROM has a better measurement property if there is evidence of adequate or very good quality from multiple studies than if there is only one study of doubtful quality. COSMIN recommends using Grading of Recommendation, Assessment, Development and Evaluation (GRADE).^10^ This is a transparent approach to assessing how confident a reviewer is with the summarised result. The quality can be rated high, moderate, low or very low.^9^

**Figure 1:**
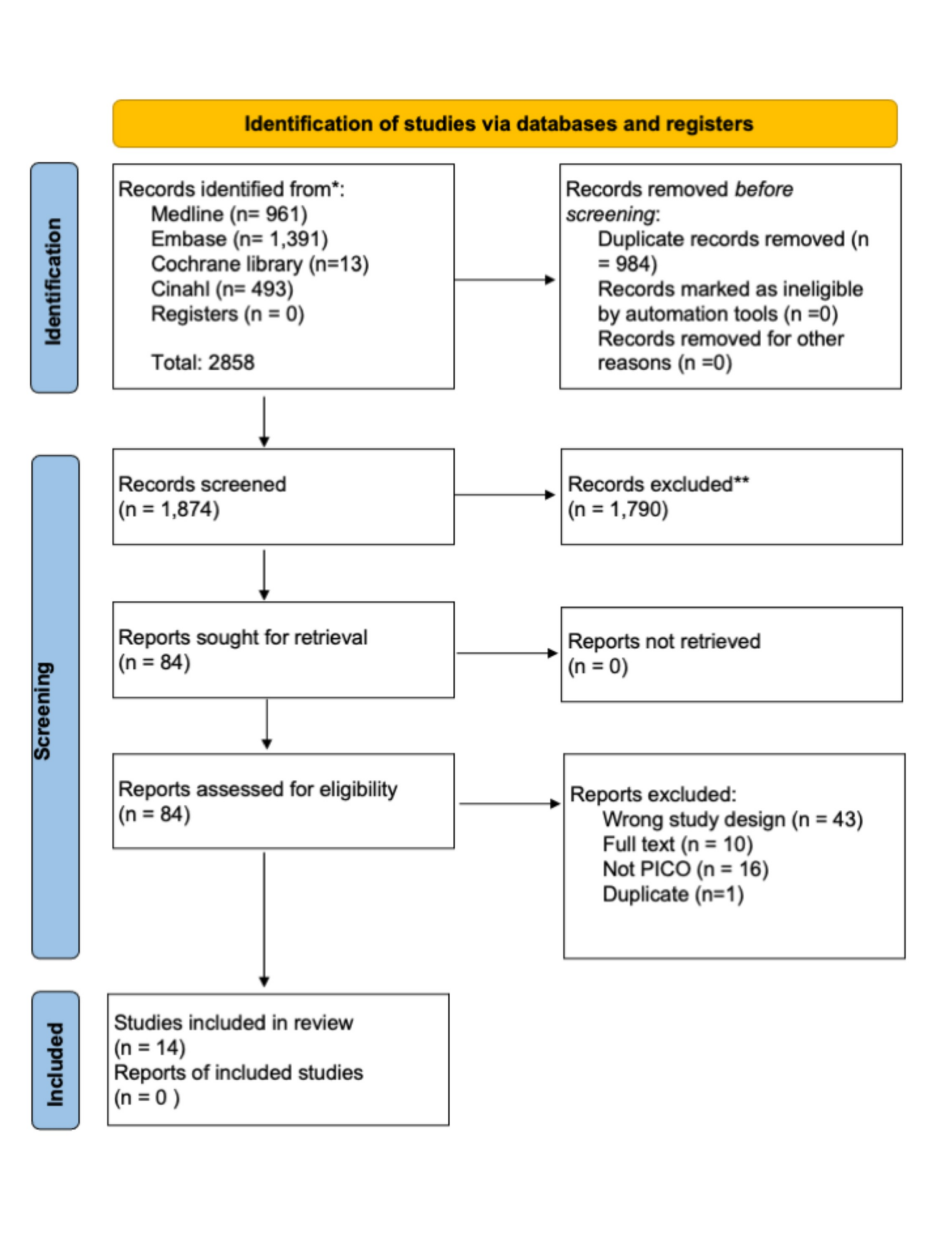
Preferred Reporting Items for Systematic Reviews and Meta-Analysis 2020 flow diagram illustrating the selection process of studies included in the systematic review. Adapted from Page *et al.*^11^ Note: this includes the updated search in this review. PICO = Population, Intervention, Comparison, Outcome.

As our aim was to evaluate the validity and reliability of PROM, we excluded an additional 13 studies that had the wrong study design. This also includes the updated search. During this process, the evaluations in COSMIN were made by the first author. The last author read and evaluated the screening, which in case of doubt was discussed between the first and last author. The screening process was reported in a Preferred Reporting Items for Systematic Reviews and Meta-Analysis (PRISMA) flowchart (figure 1).^11^

## Results

Fourteen studies with 13 different screening tools were identified (online supplemental table 3). The findings indicate that only one tool (Escape) was supported by more than one study, highlighting the need for additional data to confidently recommend any single tool as the best choice. Instead, this study presents 13 tools with potential for future use, contingent on further data collection. The aim of the present study was to evaluate the reliability and validity of screening tools used in paediatric care for identifying child maltreatment.

Each tool was evaluated based on validity and reliability requirements, following COSMIN criteria (online supplemental table 4) and the use of GRADE (online supplemental table 5).

The Escape tool was assessed in two studies, both of which were of very good quality. The remaining 12 studies varied in quality from doubtful to very good. The COSMIN checklist includes evaluation of nine measurement properties (online supplemental table 3). The best outcome is if there are many high-quality studies of the same PROM that together evaluate most of the COSMIN measurement properties. In this review, we found four studies that assessed reliability and two that assessed both reliability and content validity with adequate to very good quality. Seven of the publications did not include sensitivity or specificity. The risk of bias refers to the methodological quality of the studies, and in this review, we had to downgrade all but one of the studies due to reliance on single-study tools. Most of the single studies were of adequate quality. The ones of very good quality did not need downgrading for that reason.

The results are presented in the order of each tool’s quality.

### High quality

The studies by Louwers *et al*^12^ and Dinpanah *et al*^13^ measuring the accuracy of the Escape tool. The tool showed good sensitivity and specificity, included content validity, construct validity, internal consistency and responsiveness. The tool needs more research on reliability. As two very good studies were found, the tool’s certainty of evidence was graded high. In these studies, the Persian and Dutch versions of the Escape tool were tested in an emergency department context. Ticking one (or more) answers in the dark boxes indicates the possibility of an increased risk of child maltreatment and further action is recommended.

Shakil *et al*^14^ validated the Pediatric Hurt-Insult-Threaten-Scream-Sex (PedHITSS) tool, in this study tested in English. The tool is used in clinical settings to identify physical and sexual abuse. It was compared with the Conflict Tactics Scales Parent Child (CTSPC), the result showing that concurrent validity between the tools was strong. The quality of the study was very good and included several of the COSMIN measurement properties. The tool’s certainty of evidence was graded high because the study found was of very good quality.

### Moderate quality

Van der Put *et al*^15^ assessed the predictive validity of the Identification of Parents At Risk for Child Abuse and Neglect (IPARAN). In this study, the Dutch version of the tool was tested, which is used for parents/caregivers to fill in during home visits by healthcare professionals. The validity was better than chance, but the difference between the clinical judgement of nurses and IPARAN did not reach statistical significance. IPARAN had a sensitivity of 66.7%. The study demonstrated the highest AUC score when using both methods together. The quality of the study was adequate, as it included criterion validity and construct validity, responsiveness and content validity. IPARAN’s certainty of evidence was graded moderate because the only study found was of adequate quality.

Early Risks of Physical Abuse and Neglect Scale (ERPANS), an observation scale used by public healthcare nurses, described in Schols *et al*,^16^ which is a study validating the Dutch version of ERPANS in families with a newborn. The longitudinal study, which was of adequate quality, measured a high rate of reliability and internal consistency. It was concluded that parental mental health problems and adverse childhood experiences could be a risk factor for child maltreatment. The tool’s certainty of evidence was moderate, as only one study of adequate quality was found and no specificity, sensitivity or AUC were included.

The INTOVIAN tool was developed for use by professionals in public health settings to identify infants and toddlers at risk of emotional and physical abuse and neglect. The Escape tool was used as a reference point. The tool was tested by healthcare providers in Cyprus, Greece and Spain, with the guideline translated into Greek, Cypriot, English, Italian, Portuguese and Spanish. Feedback from professionals was collected about the items in the tool. The study was of adequate quality, assessed as moderate using GRADE due to the risk of bias and imprecision.^17^

Finding Instrument for Non-Accidental Deeds (FIND). The study by Paek *et al*^18^ aimed to develop a screening tool for child abuse and evaluate the feasibility of using it in the emergency department. The Delphi method was used in the development. The Korean version of the tool was tested for content validity. Future research needs to focus on investigating the accuracy of FIND. Our review assessed the study to be of adequate quality and the tool’s certainty of evidence was graded moderate.

The aim of Greiner *et al*^19^ was early detection of medical child abuse (MCA) and referral to a multidisciplinary team. The tool is called MCA and in this study, the English version was tested. The study showed that the tool had good construct validity but needed more testing in practical settings. The certainty of evidence was moderate, including content validity and reliability.

The Natural Language Processing tool (NLP) is an algorithm to identify high-risk injuries in electronic health record notes, designed to detect 10 specific injuries associated with physical abuse in infants. The algorithm was tested in English and had high sensitivity and specificity.^20^ Although the study includes criterion validity, there is a need for more research on validity and reliability. Because of the risk of bias, only one study was of adequate quality; therefore, this tool’s certainty of evidence was graded as moderate.

The study protocol by Sittig *et al*^21^ did not include results, although we evaluated its content as recommended. The study was of adequate quality and the Dutch version of the tool was tested. The diagnostic accuracy study, Child Abuse Inventory at Emergency Rooms, was conducted and published in 2016.^22^ The study examines whether SPUTAVAMO detects or excludes physical abuse in the emergency department and had a high false positive rate. SPUTAVAMO’s certainty of evidence was graded as moderate in this review.

Wherry *et al*^23^ report on the reliability and validity of the English version of the following two tools developed for screening symptoms of child sexual abuse (Trauma Symptom Checklist for Children Screening Form (TSCC-SF) and the Trauma Symptom Checklist for Young Children- Screening Form (TSCY-SF)). Internal consistency was very good, but only one study was found and no sensitivity, specificity or AUC were included. Alpha was calculated as 0.79–0.85. Concurrent validity was demonstrated by correlations with these two tools and two others (Children’s Attributional and Perceptual Scale, Child Behaviour Checklist). The study was of adequate quality and the tools’ certainty of evidence was moderate.

### Low quality

In Straus et al^24^ the aim was to create a parent-child version (CTSPC) of the Conflict Tactics Scale (CTS) and to compare them. The study includes a description of the conceptual and methodological approaches used. It was assessed for reliability and showed a low alpha, the same as the CTS1 tool. The English version of the tool was tested in this study. No sensitivity, specificity or AUC was included. Because only one study of adequate quality and low alpha was found, the tool’s certainty of evidence was graded as low.

Murry *et al*^25^ examined the English version of the Parents’ Support Needs Assessment (PSNA) for content validity, internal consistency, reliability and clinical usefulness. It was designed for primary care to identify families with risk factors for child maltreatment. This is a two-phase study, phase one consisted of the content validity, assessed by child maltreatment experts. Phase 2 was a pilot test of the PSNA for clinical usefulness. The result describes the PSNA as valid, reliable and having clinical usefulness. The study was of adequate quality, but phase 2 was a pilot test and therefore the tool’s certainty of evidence was downgraded to low quality.

Our review found one study of the Torso, Ear and Neck Bruising Clinical Decision Rule (TEN-4 BCDR), where the objective was to identify discriminating bruising characteristics and create a decision tool for screening children at high risk of abuse. This was a pilot study of case subjects, victims of physical abuse and control subjects, children admitted because of accidental trauma during the same time period.^26^ The English version of the tool was tested and had a high level of sensitivity and specificity for predicting abuse. The study was of adequate quality but the tool’s certainty of evidence was low.

Five tools^15^,^1720 25^ exist for identifying maltreatment in children under 3 years old, but only three are designed for children under 1 year of age.^15 16 20^ There are no tools available specifically for identification in neonatal care.

## Discussion

Screening tools can be used to detect child abuse. The tools must be valid, easy to use, succinct and amenable for use in paediatric care.^27^ Some tools have high sensitivity and cover common injuries and features of child abuse which can increase detection from 3–34%.^5^

The objective of this review was to evaluate the reliability and validity of screening tools used in paediatric care to identify child maltreatment. Eight of the studies in this review did not include sensitivity or specificity. Tools that lack sensitivity or specificity can lead to a false-positive or false-negative rating.^28^ The risk of bias refers to the methodological quality of the studies, and in this review, we needed to downgrade most of the studies due to the fact that they were the only studies of the respective tool. However, the results of most of these studies were adequate. The ones of very good quality did not need downgrading for that reason.

Most tools need a specific setting and adequate professional knowledge. None of the tools found in this review cover all types of child maltreatment; the most common is screening for physical abuse. The PSNA tool included sexual abuse, physical abuse, emotional abuse and neglect. TEN-4 BCDR, MCA, TSCC-SF/TSCY-SF, Escape, SPUTAVAMO and NLP identify specific injuries or only one form of abuse. When screening tools are used, compliance rates improve when screening all paediatric patients. Subjective screening can cause stress, making healthcare professionals, caregivers and patients feel uncomfortable and lead to caregivers becoming defensive.^29^ It is important to mention that the WHO does not recommend universal screening for violence of children who attend any type of healthcare setting, as asking all children about their experiences of violence could cause serious harm.^29^

A few (n:3) screening tools are used for assessing the risk of child maltreatment, two for healthcare providers during home visits to families with a newborn child and one for children aged <3 years. Identifying families at risk and starting early interventions and support services can lead to better outcomes for both caregiver and child. One part of the WHO’s effective and promising intervention includes early case recognition together with care of victims of child maltreatment and their families.^29^

Most of the studies assessed a few of the nine measurement properties described in the COSMIN manual, but the ones assessed could be of very good quality. Studies of PROM are more trustworthy if the measurement properties have good methodological quality. To better evaluate and recommend the screening tools found in this review, there is a need for additional studies assessing more of the COSMIN properties; thus, future research on the reliability and validity of these screening tools is required.

This review highlights several limitations in the current evidence base from a clinical perspective. Most available screening tools focus on physical abuse in emergency settings, with limited applicability to other healthcare contexts. In addition to the articles also included in Chen *et al*^6^*,* we identified six studies assessing five additional instruments. Among these, one was designed for use in emergency departments with children under 1 year of age^20^ and another targeted children under 3 years of age in primary care settings.^25^ Louwers *et al*^12^ was identified by Chen *et al*^6^ and our review. In addition, we identified another study evaluating the diagnostic accuracy of the Escape tool of very good quality.^13^ The instrument was rated as high according to GRADE in both our study and that of Chen *et al*;^6^ however, additional evaluation studies of good quality would further strengthen the evidence supporting the tool.

Although both our review and that of Chen *et al*^6^ employed COSMIN and GRADE methodologies, our study incorporates more recent evidence and places particular emphasis on tools relevant to neonatal clinical settings. Despite neonates representing a particularly vulnerable population, our findings underscore a significant evidence gap for this group.

A key challenge highlighted in this review is the contextual variability of paediatric healthcare settings. While our aim was to synthesise evidence across the full scope of paediatric care, the review confirms that most screening tools have been developed and tested in emergency departments. We acknowledge that screening tools are often highly context-specific, and that generalisability across healthcare levels may be limited. The included studies varied in terms of the healthcare professionals involved—most involved physicians or nurses, but not all studies clearly specified the level of professionals’ training of the users. This lack of detail complicates the assessment of clinical utility, as the effectiveness of a screening tool may differ depending on the professional group using it and their level of paediatric expertise.

Both Escape and PedHITSS were identified as high-quality and clinically relevant tools within this review. Their use may contribute to improving detection of child maltreatment. Escape, designed for emergency care contexts, facilitates structured clinical decision-making in cases of suspected abuse, even when presenting signs are non-specific. PedHITSS, on the other hand, enables children to self-report experiences of harm, making it particularly valuable in psychiatric and primary care settings. Notably, none of these tools specifically target the youngest age group, which was the central focus of our review.

Furthermore, the analysis of individual items across existing instruments may offer valuable insights for the development of a new, more targeted screening tool. Identifying which items are most frequently used, well-validated and applicable to specific age groups or settings could inform the construction of a context-appropriate instrument, particularly for use in early childhood or neonatal care.

Future research should prioritise the development and validation of screening instruments tailored to early childhood and neonatal care. The insights from this review may support the creation of novel tools and enhance the utility of those already in use.

### Strengths and limitations

The use of a strong search strategy in several databases with a clinical librarian is a strength in this review. We used two independent researchers for the first screening of title and abstract while four took part in the full-text screening.

Nevertheless, it is possible that we may not have identified all publications related to our aim.

It is impossible to report exactly the languages in which the tools are available.

If not described in the study, we assumed the tool was tested in the language of the country in which the study was performed.

In the initial search, we chose to include studies in English, Swedish, Norwegian and Danish. In the updated search, we decided to include only English-language studies in order to increase specificity and align with the predominant language of publication in relevant scientific journals. Furthermore, the initial search yielded no relevant studies in the Nordic languages, suggesting that the exclusion of these languages in the updated search was unlikely to affect the comprehensiveness of the review.

## Conclusions

In summary, at least 13 tools are available, but none of those identified in this review have undergone sufficient reliability and validity testing. Both the Escape and PedHITSS tools are high-quality and clinically useful, but further testing in healthcare settings is needed for greater certainty, as only a few studies have been conducted on these tools. In line with our primary focus on infants and toddlers, we also aim to develop or identify instruments specifically designed for these age groups.

## Supplementary material

10.1136/bmjopen-2025-101721 online supplemental file 1

10.1136/bmjopen-2025-101721 online supplemental file 2

## Data Availability

All data relevant to the study are included in the article or uploaded as supplementary information.
