# Supplementary material for: Screening tools used by paediatric healthcare providers to identify child maltreatment by parents or caregivers: a systematic review
Source: BMJ Open. 2025 Aug 4;15(8):e101721. doi: 10.1136/bmjopen-2025-101721 (PMC12323525; doi:10.1136/bmjopen-2025-101721)
Supplement: online supplemental file 1 [file bmjopen-15-8-s001.docx]

**Table 1a.**

**Database:** Ovid MEDLINE(R) ALL (OvidSP)

**Date:** 2023-11-01

**No of results:** 822 ref

| **#** | **Searches** | **Results** |
| --- | --- | --- |
| 1 | exp Infant/ | 1258464 |
| 2 | exp Child/ | 2167861 |
| 3 | exp Adolescent/ | 2224648 |
| 4 | (child* or infant* or baby or babies or preterm* or neonat* or newborn* or toddler* or adolescen* or teen*).ab,kf,ti. | 2564223 |
| 5 | 1 or 2 or 3 | 3989728 |
| 6 | 4 or 5 | 4719786 |
| 7 | (maltreat* or neglect* or mistreat* or abus*).ab,kf,ti. | 242904 |
| 8 | 6 and 7 | 82761 |
| 9 | exp Child Abuse/ | 34816 |
| 10 | 8 or 9 | 92170 |
| 11 | (Healthcare or hospital* or clinic* or ward*).ab,kf,ti. | 6589618 |
| 12 | (detect* or screening).ab,kf,ti. | 3358434 |
| 13 | (instrument* or tool* or scale).ab,kf,ti. | 2202813 |
| 14 | 12 and 13 | 373730 |
| 15 | 10 and 11 and 14 | 869 |
| 16 | (animals not (animals and humans)).sh. | 5130614 |
| 17 | 15 not 16 | 869 |
| 18 | (comment or editorial or letter).pt. | 2200680 |
| 19 | 17 not 18 | 868 |
| **20** | **limit 19 to (danish or english or norwegian or swedish)** | **822** |

**exp/** = term from the Medline controlled vocabulary, including terms found below this term in the MeSH hierarchy

**/** = term from the Medline controlled vocabulary, does not include terms found below this term in the MeSH hierarchy

**.ti,ab,kf**. = title, abstract and author keywords

***** = truncation of word for alternate endings

**Table 1 b.**

**Database:** Embase <1974 to 2023 October 31>

**Date:** 2023-11-01

**No of results:** 1160 ref

| **#** | **Searches** | **Results** |
| --- | --- | --- |
| 1 | exp infant/ | 1143793 |
| 2 | exp child/ | 3112482 |
| 3 | exp adolescent/ | 1787127 |
| 4 | (child* or infant* or baby or babies or preterm* or neonat* or newborn* or toddler* or adolescen* or teen*).ab,kf,ti. | 3153022 |
| 5 | 1 or 2 or 3 | 3978117 |
| 6 | 4 or 5 | 4828643 |
| 7 | (maltreat* or neglect* or mistreat* or abus*).ab,kf,ti. | 312832 |
| 8 | 6 and 7 | 95869 |
| 9 | exp child abuse/ | 45476 |
| 10 | 8 or 9 | 108278 |
| 11 | (Healthcare or hospital* or clinic* or ward*).ab,kf,ti. | 9377567 |
| 12 | (detect* or screening).ab,kf,ti. | 4348366 |
| 13 | (instrument* or tool* or scale).ab,kf,ti. | 2887472 |
| 14 | 12 and 13 | 517722 |
| 15 | 10 and 11 and 14 | 1223 |
| 16 | animal/ not (animal/ and human/) | 1201096 |
| 17 | 15 not 16 | 1223 |
| 18 | (comment or editorial or letter).pt. | 2077431 |
| 19 | 17 not 18 | 1222 |
| **20** | **limit 19 to (danish or english or norwegian or swedish)** | **1160** |

**exp/** = term from the emtree controlled vocabulary, including terms found below this term in the hierarchy

**/** = term from the emtree controlled vocabulary, does not include terms found below this term in the hierarchy

**.ti,ab,kf**. = title, abstract and author keywords

***** = truncation of word for alternate endings

**Table 1 c.**

**Database:** The Cochrane Library
**Date:** 2023-11-01
**No of results:** 10 ref

*Cochrane reviews: 1
Cochrane protocols: 0
Trials: 9
Editorials: 0
Special collections: 0
Clinical answers: 0*

| **ID** | **Search** | **Hits** |
| --- | --- | --- |
| #1 | MeSH descriptor: [Infant] explode all trees | 42134 |
| #2 | MeSH descriptor: [Child] explode all trees | 78764 |
| #3 | MeSH descriptor: [Adolescent] explode all trees | 126020 |
| #4 | (child* or infant* or baby or babies or preterm* or neonat* or newborn* or toddler* or adolescen* or teen*):ti,ab,kw | 348683 |
| #5 | #1 OR #2 OR #3 | 191036 |
| #6 | #4 OR #5 | 348683 |
| #7 | (maltreat* or neglect* or mistreat* or abus*):ti,ab,kw | 18374 |
| #8 | #6 AND #7 | 5742 |
| #9 | MeSH descriptor: [Child Abuse] explode all trees | 716 |
| #10 | #8 AND #9 | 716 |
| #11 | (Healthcare or hospital* or clinic* or ward*):ti,ab,kw | 1130309 |
| #12 | (detect* or screening):ti,ab,kw | 165965 |
| #13 | (instrument* or tool* or scale):ti,ab,kw | 319258 |
| #14 | #12 AND #13 | 35156 |
| **#15** | **#10 AND #11 AND #14** | **10** |

**Table 1 d.**

**Database:** Cinahl

**Date:** 2023-11-01

**No of results:** 454 ref

| **#** | **Query** | **Results** |
| --- | --- | --- |
| **S16** | **S10 AND S11 AND S14 Narrow by Language: - english** | **454** |
| S15 | S10 AND S11 AND S14 | 462 |
| S14 | S12 AND S13 | 70,15 |
| S13 | TI ( instrument* or tool* or scale ) OR AB ( instrument* or tool* or scale ) | 572,002 |
| S12 | TI ( detect* or screening ) OR AB ( detect* or screening ) | 430,308 |
| S11 | TI ( Healthcare or hospital* or clinic* or ward* ) OR AB ( Healthcare or hospital* or clinic* or ward* ) | 2,003,162 |
| S10 | S8 OR S9 | 47,775 |
| S9 | (MH "Child Abuse+") | 24,055 |
| S8 | S6 AND S7 | 38,949 |
| S7 | TI ( maltreat* or neglect* or mistreat* or abus* ) OR AB ( maltreat* or neglect* or mistreat* or abus* ) | 91,39 |
| S6 | S4 OR S5 | 1,388,932 |
| S5 | S1 OR S2 OR S3 | 1,109,866 |
| S4 | TI ( child* or infant* or baby or babies or preterm* or neonat* or newborn* or toddler* or adolescen* or teen* ) OR AB ( child* or infant* or baby or babies or preterm* or neonat* or newborn* or toddler* or adolescen* or teen* ) | 877,839 |
| S3 | (MH "Adolescence+") | 603,927 |
| S2 | (MH "Child+") | 754,058 |
| S1 | (MH "Infant+") | 286,318 |

**MH** = term from the Cinahl controlled vocabulary, including terms found below this term in the hierarchy

**.TI = title**

**AB = abstract**

***** = truncation of word for alternate endings

**Table 2a. Updated search**

**Database:** Ovid MEDLINE(R) ALL (OvidSP)

**Date:** 2025-06-24

**No of results:** 139 ref

| 1 | exp Infant/ | 1312395 |
| --- | --- | --- |
| 2 | exp Child/ | 2273578 |
| 3 | exp Adolescent/ | 2334689 |
| 4 | (child* or infant* or baby or babies or preterm* or neonat* or newborn* or toddler* or adolescen* or teen*).ab,kf,ti. | 2766771 |
| 5 | 1 or 2 or 3 | 4179671 |
| 6 | 4 or 5 | 4993717 |
| 7 | (maltreat* or neglect* or mistreat* or abus*).ab,kf,ti. | 264221 |
| 8 | 6 and 7 | 89539 |
| 9 | exp Child Abuse/ | 36748 |
| 10 | 8 or 9 | 99082 |
| 11 | (Healthcare or hospital* or clinic* or ward*).ab,kf,ti. | 7351807 |
| 12 | (detect* or screening).ab,kf,ti. | 3683210 |
| 13 | (instrument* or tool* or scale).ab,kf,ti. | 2535604 |
| 14 | 12 and 13 | 434800 |
| 15 | 10 and 11 and 14 | 972 |
| 16 | (animals not (animals and humans)).sh. | 5315154 |
| 17 | 15 not 16 | 972 |
| 18 | (comment or editorial or letter).pt. | 2334477 |
| 19 | 17 not 18 | 971 |
| 20 | limit 19 to (yr="2023 -Current" and english) | 139 |

**Table 2 b. Updated search**

**Database:** Embase

**Date:** 2025-06-23

**No of results:** 231 ref

| 1 | exp infant/ | 1238522 |
| --- | --- | --- |
| 2 | exp child/ | 3423964 |
| 3 | exp adolescent/ | 1972337 |
| 4 | (child* or infant* or baby or babies or preterm* or neonat* or newborn* or toddler* or adolescen* or teen*).ab,kf,ti. | 3486586 |
| 5 | 1 or 2 or 3 | 4370576 |
| 6 | 4 or 5 | 5292054 |
| 7 | (maltreat* or neglect* or mistreat* or abus*).ab,kf,ti. | 345286 |
| 8 | 6 and 7 | 105685 |
| 9 | exp child abuse/ | 49177 |
| 10 | 8 or 9 | 118657 |
| 11 | (Healthcare or hospital* or clinic* or ward*).ab,kf,ti. | 10731570 |
| 12 | (detect* or screening).ab,kf,ti. | 4839689 |
| 13 | (instrument* or tool* or scale).ab,kf,ti. | 3361668 |
| 14 | 12 and 13 | 612845 |
| 15 | 10 and 11 and 14 | 1564 |
| 16 | animal/ not (animal/ and human/) | 1257170 |
| 17 | 15 not 16 | 1564 |
| 18 | (comment or editorial or letter).pt. | 2203684 |
| 19 | 17 not 18 | 1563 |
| 20 | limit 19 to (english and yr="2023 -Current") | 231 |

**Table 2c. Updated search**

**Database:** The Cochrane Library
**Date:** 2025-06-23
**No of results:** 3 ref

| ID | Search | Hits |
| --- | --- | --- |
| #1 | MeSH descriptor: [Infant] explode all trees | 45762 |
| #2 | MeSH descriptor: [Child] explode all trees | 82406 |
| #3 | MeSH descriptor: [Adolescent] explode all trees | 137104 |
| #4 | (child* or infant* or baby or babies or preterm* or neonat* or newborn* or toddler* or adolescen* or teen*):ti,ab,kw | 383635 |
| #5 | #1 OR #2 OR #3 | 204921 |
| #6 | #4 OR #5 | 383635 |
| #7 | (maltreat* or neglect* or mistreat* or abus*):ti,ab,kw | 19715 |
| #8 | #6 AND #7 | 6258 |
| #9 | MeSH descriptor: [Child Abuse] explode all trees | 795 |
| #10 | #8 AND #9 | 795 |
| #11 | (Healthcare or hospital* or clinic* or ward*):ti,ab,kw | 1251753 |
| #12 | (detect* or screening):ti,ab,kw | 184259 |
| #13 | (instrument* or tool* or scale):ti,ab,kw | 372764 |
| #14 | #12 AND #13 | 40693 |
| #15 | #10 AND #11 AND #14 | 14 |
|  | Limit #15 to 2023-2025 | 3 |

**Table 2 d. Updated search.**

**Database:** Cinahl

**Date:** 2025-06-24

**No of results:** 39 ref

| S17 | S10 AND S11 AND S14 Narrow by Language: - english,  Limiters - Publication Date: 20230101-20251231 | 39 |
| --- | --- | --- |
| S16 | S10 AND S11 AND S14 Narrow by Language: - english | 474 |
| S15 | S10 AND S11 AND S14 | 483 |
| S14 | S12 AND S13 | 75,378 |
| S13 | TI ( instrument* or tool* or scale ) OR AB ( instrument* or tool* or scale ) | 612,029 |
| S12 | TI ( detect* or screening ) OR AB ( detect* or screening ) | 449,754 |
| S11 | TI ( Healthcare or hospital* or clinic* or ward* ) OR AB ( Healthcare or hospital* or clinic* or ward* ) | 2,065,741 |
| S10 | S8 OR S9 | 50,13 |
| S9 | (MH "Child Abuse+") | 25,525 |
| S8 | S6 AND S7 | 40,64 |
| S7 | TI ( maltreat* or neglect* or mistreat* or abus* ) OR AB ( maltreat* or neglect* or mistreat* or abus* ) | 92,15 |
| S6 | S4 OR S5 | 1,461,132 |
| S5 | S1 OR S2 OR S3 | 1,162,906 |
| S4 | TI ( child* or infant* or baby or babies or preterm* or neonat* or newborn* or toddler* or adolescen* or teen* ) OR AB ( child* or infant* or baby or babies or preterm* or neonat* or newborn* or toddler* or adolescen* or teen* ) | 918,936 |
| S3 | (MH "Adolescence+") | 637,189 |
| S2 | (MH "Child+") | 784,44 |
| S1 | (MH "Infant+") | 294,326 |
