# Supplementary material for: Screening tools used by paediatric healthcare providers to identify child maltreatment by parents or caregivers: a systematic review
Source: BMJ Open. 2025 Aug 4;15(8):e101721. doi: 10.1136/bmjopen-2025-101721 (PMC12323525; doi:10.1136/bmjopen-2025-101721)
Supplement: online supplemental file 2 [file bmjopen-15-8-s002.docx]

**Table 3.** Selected studies and characteristics.

| **Reference** | **PROM** | **Country** | **Inclusion criteria** | **Sample size** | **Child maltreatment** | **Setting** | **No. Of items** | **Characteristics of tool** | **Sensitivity (95% CI)** | **Specificity (95% CI)** | **AUC (95% CI)** | **Study** |
| --- | --- | --- | --- | --- | --- | --- | --- | --- | --- | --- | --- | --- |
| Straus et al, 1998 | CTSPC | United states | <18 years old | 1000 | Physical abuse and neglect | Home enviroment | 27 | Parental behavior. Items: Nonviolent discipline (4 items), Psychological aggression (5 items), Physical assault (13 items), neglect (5 items). | - | - | - | Description of the conceptual and methodological approaches used and psychometric data for 1000 children. Low alpha. |
| Schols et al, 2019 | ERPANS | Netherlands | <1 years old, home visits | 1257 | Physical abuse and neglect. Home visits. | Home enviroment | 31 | Nurse rated observation scale. 4-point response format | - | - | - | Independent prospective validation of the Erpans in a Dutch community sample of families with a newborn. |
| Dinpanah et al, 2017 | Escape | Iran | <16 years old | 6120 | Physical abuse | Emergency department | 6 | Yes/no questions. Used in Emergency department for professionals. | 100 (87.6 – 100) | 98.3 (97.9 – 98.6) | 99.2 (98.9 – 99.4) | Evaluate the diagnostic accuracy of Escape instrument in triage of at-risk cases of child maltreatment presenting to the emergency department. |
| Louwers et al, 2014 | Escape | Netherlands | <18 years old | 18,275 | Physical abuse | Emergency department | 6 | Yes/no questions. Used in Emergency department for professionals. | 80 [67.0, 89.0] | 98 | - | Measure the accuracy of ESCAPE for detection of potential child abuse used in the emergency department. |
| Paek et al, 2018 | FIND | Korea | <14 years old | 3855 | Physical abuse, neglect | Emergency department | 8 | Yes/no questions. By healthcare providers | - | - | - | Development. Delphi method. Feasibility test. Testing ITEMS. |
| Espeleta et al, 2016 | INTOVIAN | Spain, Cyprus, Greece | <3 years old | 219 | Physical abuse, emotional abuse, neglect. | Public healthcare setting | 6 | Yes/no questions. Used by healthcare providers. Public healthcare sector. Infants and toddlers. | - | - | - | Professionals, three-step process, developing Intovian. |
| Van der Put et al, 2017 | IPARAN | Netherlands | < 1 years old, home visits by nurses. | 4692 | Child abuse and neglect. Home visits. | Home enviroment | 16 | Responses: Always/often/sometimes/never OR yes/no. Questionnaire for parents or caregivers. | 66,7 | 77,4 | 72 [59.3, 84.7] | Predictive validity of IPARAN. Examine whether combining actuarial and clinical methods leads to an improvement of the predictive validity. |
| Greiner et al, 2013 | MCA | United states | <17 years old | 408 | MCA (Medical Child Abuse) | Hospital | 46 | Characteristics of the caregiver (7 questions) child (24 questions) symptoms (Apnéa (6 questions) vomiting and diarrhea (4 questions) seizures (5 questions)) | 94,7 | 95,6 | 95% CI and ROC | Records reviewed. Evaluated the association of each item to determine an OR and CI for association with case status in an unmatched analysis. 95% CI calculated on the ITEMS. |
| Tiyyagura et al, 2022 | NLP algoritm | United states | <1 years old | 1344 | Physical abuse | Emergency department | - | Natural language understanding platform that provides the foundation for speech recognition. | 92,7 (79.0–98.1) | 98,1 (97.1–98.7) | - | Examine nursing, provider, and social work notes in the ED to identify high-risk injuries concerning for abuse in children <1 years old. |
| Shakil et al, 2018 | Ped-HITSS | United States | <12 years old | 422 | Physical abuse, sexual abuse. | Clinic | 5 | Parents asked to complete PED-HITSS and CTSPC questionnaires. 5 Point scale. | Counted but no number in text | Counted but no number in text | 85 (81.0, 89.0) | Concurrent validity was strong between PED-HITSS and CTSPC. |
| Murry et al, 2014 | PSNA | United States | <3 years old | 10 | Physical abuse, sexual abuse, emotional abuse, neglect. | Primary care | 20 | Yes/no, for providers to administrate. Four risk categories. a) demographic factors b) parental characteristics c) child characteristics d) contextual factors. | - | - | - | Two phases. Phase one Content validity by child maltreatment experts. CV-score. Reliability score. Phase two pilot test of the PSNA. Pilot. |
| Sittig et al, 2011 | SPUTAVAMO | Netherlands | <7 years old | 4290 | Physical abuse | Emergency department | 6 | Yes/no questions. By healthcare providers. Emergency departement. | - | - | - | Whether Sputavamo detects or excludes physical abuse in ER. High false positive rate. |
| Pierce et al, 2010 | TEN-4 BCDR | United States | <4 years old | 95 | Physical abuse, bruising characteristics | PICU | - | Bruising location. TEN (Torso, Ear and Neck) child < 4 years of age, and bruising in any area on infant <4 months. Used by healthcare providers. | 97% | 84% | - | Case-control study. Victims of physical abuse and control subjects were children admitted to PICU because of accidental trauma during the same time. |
| Wherry et al, 2018 | TSCC-SF TSCYC-SF | United States | TSCC-SF ages 8-16. TSCYC-SF ages 3-12 | 200 | Sexual abuse. | Outpatient setting | 20 | Two subscales, one for nonsexual symptoms and one for sexual symptoms. By children and caretakers depending of the age of the child. | - | - | - | Reliability and validity for two measures developed for screening child sexual abuse. Alpha 0,79–0,85. Correlations with the TSCC and TSCYC. |

**Table 4.** COSMIN (Consensus-based Standards for the Selection of Health Measurement Instruments) checklist.

| **Study** | **Tool** | **Internal Consistency** | **Reliability** | **Measurement Error** | **Content Validity** | **Structural Validity** | **Hypotheses Testing for construct validity** | **Criterion Validity** | **Responsive-ness** | **Cross-Culture Validity** |
| --- | --- | --- | --- | --- | --- | --- | --- | --- | --- | --- |
| Straus et al, 1998 | CTSPC | Very good/ - |  |  |  |  |  |  |  |  |
| Schols et al, 2019 | ERPANS | Very good/ + | Adequate/+ | Adequate/? |  | Adequate/? |  |  |  |  |
| Dinpanah et al, 2017 | Escape |  |  |  |  |  |  | Very good/+ | Adequate/+ |  |
| Louwers et al, 2014 | Escape | Very good/+ |  | Adequate/? | Adequate/+ |  | Adequate/+ | Very good/? | Very good/+ |  |
| Paek et al, 2018 | FIND |  |  |  | Adequate/+ |  | Adequate/+ |  |  |  |
| Espeleta et al, 2016 | INTOVIAN | Very good/+ | Adequate/? |  | Adequate/+ |  | Adequate/+ |  |  |  |
| Van der Put et al, 2017 | IPARAN |  |  |  |  |  | Adequate/+ | Very good/- | Very good/+ |  |
| Greiner et al, 2013 | MCA |  |  |  |  |  | Adequate/+ | Very good/+ | Very good/+ |  |
| Tiyyagura et al, 2022 | NLP algoritm |  |  |  |  |  |  | Very good/? |  |  |
| Shakil et al, 2018 | Ped-HITSS | Very good/ + | Adequate/+ | Adequate/? |  | Adequate/- | Adequate/+ | Very good/+ | Adequate/+ |  |
| Murry et al, 2014 | PSNA | Very good/ + | Very good/+ |  | Adequate/+ |  |  |  |  |  |
| Sittig et al, 2011 | SPUTAVAMO |  |  |  | Very good/+ |  | Verry good/+ | Adequate/? |  |  |
| Pierce et al, 2010 | TEN-4 BCDR |  |  | Adequate/? |  |  | Adequate/+ | Very good/? | Adequate/+ |  |
| Wherry et al, 2018 | TSCC-SF TSCYC-SF | Very good/ + |  |  |  |  |  |  |  |  |

**Table 5.** GRADE Summary of Findings on Screening Tools for Child Abuse

| **Reference** | **PROM** | **Risk of bias** | **Inconsistency** | **Indirectness** | **Imprecision** | **Publication bias** | **Study**** | **Quality of evidence PROM** |
| --- | --- | --- | --- | --- | --- | --- | --- | --- |
| Straus et al 1998 | CTSPC | Serious*5 | Not serious | Not serious | Serious*4*1 | Undetected | Doubtful | Low, due to risk of bias and imprecision |
| Schols et al 2019 | ERPANS | Serious*5 | Not serious | Not serious | Serious*1 | Undetected | Adequate | Moderate, due to risk of bias and imprecision |
| Dinpanah et al 2017 | Escape | Not serious | Not serious | Not serious | Not serious | Undetected | Very good | High |
| Louwres et al 2014 | Escape | Not serious | Not serious | Not serious | Not serious | Undetected | Very good | High |
| Paek et al 2018 | FIND | Serious*5 | Not serious | Not serious | Serious*1 | Undetected | Adequate | Moderate, due to risk of bias and imprecision |
| Espeleta et al 2016 | INTOVIAN | Serious*5 | Not serious | Not serious | Serious*1 | Undetected | Adequate | Moderate, due to risk of bias and imprecision. |
| Van der Put et al 2017 | IPARAN | Serious*5 | Not serious | Not serious | Serious*2 | Undetected | Adequate | Moderate, due to risk of bias and imprecision. |
| Greiner et al 2013 | MCA | Serious*5 | Not serious | Not serious | Not serious | Undetected | Adequate | Moderate, due to risk of bias |
| Tiyyagura et al 2022 | NLP algoritm | Serious*5 | Not serious | Not serious | Not serious | Undetected | Adequate | Moderate, due to risk of bias |
| Shakil et al 2018 | Ped-HITSS | Not serious | Not serious | Not serious | Not serious | Undetected | Very good | High |
| Murry et al 2014 | PSNA | Serious*5*6 | Not serious | Not serious | Serious*1 | Undetected | Adequate | Low, Due to risk of bias and imprecision |
| Sittig et al 2011 | SPUTAVAMO | Not serious | Not serious | Not serious | Serious*1 | Undetected | Adequate | Moderate, due to imprecision |
| Pierce et al 2010 | TEN-4 BCDR | Serious *5*6 | Not serious | Not serious | Serious*1 | Undetected | Adequate | Low, due to risk of bias and imprecision |
| Wherry et al 2018 | TSCC-SF TSCYC-SF | Serious*5 | Not serious | Not serious | Serious*1 | Undetected | Adequate | Moderate, due to risk of bias and imprecision |

**Grading studies using COSMIN

*1 Studies were insufficient to provide screening validity of AUC or sensitivity or specificity without 95% CI, *2 The study shows low AUC, specificity or sensitivity

*4 The study shows low alpha, *5 One study of adequate quality, *6. Low number participants included in study (e.g., pilot study), *7 One study of doubtful quality.
