# Supplementary material for: Screening tools used by paediatric healthcare providers to identify child maltreatment by parents or caregivers: a systematic review
Source: BMJ Open. 2025 Aug 4;15(8):e101721. doi: 10.1136/bmjopen-2025-101721 (PMC12323525; doi:10.1136/bmjopen-2025-101721)
Supplement: Supplementary file 3 [file bmjopen-2025-101721.reviewer_comments.pdf]

PEER REVIEW HISTORY

BMJ Open publishes all reviews undertaken for accepted manuscripts. Reviewers are asked to complete a checklist review form and are provided with free text boxes to elaborate on their assessment. These free text comments are reproduced below.

ARTICLE DETAILS

Title (Provisional)

Screening Tools Used by Paediatric Healthcare Providers to identify Child Maltreatment by Parents or Caregivers: A Systematic Review

Authors

Ejnell, Josefine; Wigert, Helena; Patriksson, Katarina; Nilsson, Stefan

VERSION 1 - REVIEW

Reviewer 1

Name Raman, Shanti

Affiliation University of New South Wales School of Women's and Children's Health

Date 20-Apr-2025

COI Not applicable. But I have already provided a previous review of this submission for BMJPO

General

I have previously reviewed the same manuscript for BMJPO. Not many revisions have been made to the subsequent submission to BMJ Open. This is an important topic, but one that has been looked at not infrequently. I still think having a broad area of enquiry- that is all of paediatric healthcare- is a problem, because screening tools are usually context specific, so those that are used for acute frontline healthcare settings, may not be useful for others. This is a global review, but there was an inclusion of studies published in Swedish, Danish or Norwegian- as well as English, there needs to be some justification of this slant. Perhaps 'paediatric care' in Scandinavian countries is a bit more aligned than elsewhere in the world? Authors do say that mostly this refers to doctors and nurses and that the primary settings for the review relate to pediatric hospital wards and emergency rooms. I note however that the settings of the included studies involve 'home', primary care', 'clinic' as well as ED, PICU, Outpatient clinic in hospital- this is a very wide range of settings, and the tools used necessarily will vary. Finally, the clinicians involved are also not specified- what level, are they paediatric specialised workforce, nurses, doctors, social workers? So this needs to be fleshed out a bit more, than this: The aim of this review is to update (?broaden search terms from 2022 Chen review) the search for studies on screening tools used in paediatric care *(define and by which category of health professionals)*) to identify child maltreatment *(?parameters)* and to evaluate their validity and reliability. Given that the authors are clearly not interested in re-doing this systematic review

write-up, I would suggest that some attempt is made to separate out hospital settings versus community-based settings, ie home and primary care, a sub-group analysis perhaps would be worthwhile.

Results of a systematic review of such detail- should actually provide clinically useful information- and I am glad that the authors have made a call about the Escape tool and the PedHITSS, which were of high quality and low risk of bias. What potential can these tools have for clinical use in frontline settings?

I cannot fault the methods of this systematic review, I still think however, that the clinical lens and thus the utility from this review is missing. It might just be that the plethora of tools that have been trialled in various clinical settings cannot be generalisable, or indeed that 'screening' for child maltreatment, may not be a useful concept. Rather perhaps comprehensive assessment of children in all acute frontline settings, would be more useful.

Discussion- Conclusions

Provide more cogent exploration of the limitations of this review- especially from a clinical perspective, as I have outlined. And provide clinically useful analysis and recommendations, in particular, how much more does this review add to what is already known from the Chen review, which stated: "future research should focus on the use of screening tools in the healthcare system to identify effective screening interventions that may help healthcare providers identify child abuse cases as early as possible". In other words, what is the justification for updating this systematic review from Chen's 2022 review?

Reviewer 2

Name Chang, Hsin-Yi

Affiliation National Cheng Kung University College of Medicine, Department of Nursing

Date 19-May-2025

COI None

Thank you for the opportunity to review this manuscript. This manuscript aims to evaluate the psychometric properties of child maltreatment screening tools in pediatric healthcare settings by using the COSMIN checklist. However, the significance of this study, along with certain elements of the results and discussion, raises concerns regarding its suitability for publication. There are some points I would make:

Introduction:

1. The introduction section would benefit from a clearer articulation of the study's significance, particularly regarding the rationale for conducting an updated review of the screening tools for child abuse. A critical analysis of the current state of science in this topic is essential to address gaps in the literature and argue for the necessity and importance of this systematic review (i.e., comparing existing screening tools and selecting well-validated ones for healthcare providers). Moreover, emphasizing existing research gaps, such as the

weaknesses of the previous review by Chen et al. (2022), or newly developed tools in recent years, would strengthen the justification for this updated review and underscore its contribution to the field.

Methods:

1. Design: p. 7, lines 14 – 23, the authors stated that this review was focused on young children but spanned the children's age from 0 to 18 years to prevent missing cases. Though it is reasonable to extend the age range during the literature search, it is recommended to stay focused on the targeted population, especially in the results and discussion sections, to highlight and discuss the quality and appropriateness of the existing child abuse screening tools for young children.

2. Search strategy:

(1) Table 1 is recommended to be placed in the appendix.

(2) Table 1d is the search strategy used for the CINAHL database, which was not mentioned in the text.

3. Search outcome: Figure 1 PRISMA flowchart recommends using the PRISMA 2020 flow diagram.

Results & discussion:

1. Results: In addition to categorizing the screening tools by their quality levels, it is recommended to identify which tools are most applicable for use with young children, in alignment with the specific focus of this study. Highlighting age-appropriate tools will enhance the practical value and relevance of the findings.

2. Discussion: It is recommended to discuss the similarities and differences with the previous systematic review by Chen et al. (2022) to underline the strengths and significance of this review.

Overall, the manuscript would benefit from reorganization and restructuring, particularly in the introduction and discussion sections.

VERSION 1 - AUTHOR RESPONSE

Reviewer 1: Dr. Shanti Raman, University of New South Wales School of Women's and Children's Health, South Western Sydney Local Health District

This is a global review, but there was an inclusion of studies published in Swedish, Danish or Norwegian- as well as English, there needs to be some justification of this slant. Perhaps 'paediatric care' in Scandinavian countries is a bit more aligned than elsewhere in the world?

Response: Thank you for raising this important point. Including Swedish, Norwegian, and Danish did not yield any additional studies. Therefore, we chose to limit the updated search to studies published in English, which also includes studies from the Nordic countries, as these are typically published in English.

Page: 5, 11, section 7.

Authors do say that mostly this refers to doctors and nurses and that the primary settings for the review relate to pediatric hospital wards and emergency rooms. I note however that the settings of the included studies involve 'home', primary care', 'clinic' as well as ED, PICU, Outpatient clinic in hospital- this is a very wide range of settings, and the tools used necessarily will vary.

Response: Thank you for this important observation. We agree that the included studies span a wide range of clinical and community settings, including home environments, primary care, clinics, emergency departments, PICUs, and outpatient hospital clinics. This diversity reflects the varied contexts in which child maltreatment may be identified and screened for.

While our primary focus was on tools applicable in paediatric hospital settings—particularly neonatal care—we recognize that several tools were developed and validated in broader or differing contexts. We have now clarified this in the manuscript, acknowledging the variation in study settings and the potential implications this may have on the applicability of specific tools in neonatal hospital care.

Page. 11, section 2.

Finally, the clinicians involved are also not specified- what level, are they paediatric specialised workforce, nurses, doctors, social workers? So this needs to be fleshed out a bit more, than this: The aim of this review is to update (?broaden search terms from 2022 Chen review) the search for studies on screening tools used in paediatric care (define and by which category of health professionals)) to identify child maltreatment (?parameters) and to evaluate their validity and reliability. Given that the authors are clearly not interested in re-doing this systematic review write-up, I would suggest that some attempt is made to separate out hospital settings versus community-based settings, ie home and primary care, a sub-group analysis perhaps would be worthwhile.

Response:
Thank you for this important observation. We have now clarified in the Methods section which types of healthcare professionals were involved where reported, including physicians and nurses. We have also added a part in the discussion- section.

Page. 4, section 4,5. Page. 11.

Results of a systematic review of such detail- should actually provide clinically useful information- and I am glad that the authors have made a call about the Escape tool and the PedHITSS, which were of high quality and low risk of bias. What potential can these tools have for clinical use in frontline settings?

Response:

Thank you for your point, We have added and clarified in page. 11.

I cannot fault the methods of this systematic review, I still think however, that the clinical lens and thus the utility from this review is missing. It might just be that the plethora of tools that have been trialled in various clinical settings cannot be generalisable, or indeed that 'screening' for child maltreatment, may not be a useful concept. Rather perhaps comprehensive assessment of children in all acute frontline settings, would be more useful. Discussion- Conclusions Provide more cogent exploration of the limitations of this review- especially from a clinical perspective, as I have outlined. And provide clinically useful analysis and recommendations, in particular, how much more does this review add to what is already known from the Chen review, which stated: "future research should focus on the use of screening tools in the healthcare system to identify effective screening interventions that may help healthcare providers identify child abuse cases as early as possible". In other words, what is the justification for updating this systematic review from Chen's 2022 review?

Response:

Thank you for your comments — we appreciate them and understand that further clarification is needed. More information in the discussion-section.

Furthermore, the analysis of individual items across existing instruments may offer valuable insights for the development of a new, more targeted screening tool. Identifying which items are most frequently used, well-validated, and applicable to specific age groups or settings could inform the construction of a context-appropriate instrument, particularly for use in early childhood or neonatal care.

Page 11

Reviewer 2:
Dr. Hsin-Yi Chang, National Cheng Kung University College of Medicine

---

Conclusion

We once again thank the reviewers for their insightful comments, which have strengthened our work. We look forward to your further evaluation of our manuscript.

Introduction:
1. The introduction section would benefit from a clearer articulation of the study's significance, particularly regarding the rationale for conducting an updated review of the screening tools for child abuse. A critical analysis of the current state of science in this topic is essential to address gaps in the literature and argue for the necessity and importance of this systematic review (i.e., comparing existing screening tools and selecting well-validated ones for healthcare providers). Moreover, emphasizing existing research gaps, such as the weaknesses of the previous review by Chen et al.

(2022), or newly developed tools in recent years, would strengthen the justification for this updated review and underscore its contribution to the field.

Response
Thank you for this comment, we have now made the significance clearer in the introduction section:

In the Swedish healthcare context, particularly within nursing and care for young children, it is crucial to utilise evidence-based screening instruments that are both specific and sensitive in detecting suspected child maltreatment. Such tools should have a high number needed to treat (NNT), effectively identifying families genuinely in need of intervention, while maintaining a low number needed to harm (NNH) to avoid falsely accusing innocent families. This precision is essential to foster trust between families and healthcare professionals, ensure that resources are appropriately allocated, and prioritise the child's safety without causing undue harm.
An updated systematic review is warranted to assess whether new studies have emerged that further evaluate the instruments identified by Chen et al. (2022), or if any new screening tools have been developed—particularly those applicable to neonatal care settings. The present review serves as a foundation for the implementation of a tool designed to support specialist- physicians and nurses in Swedish neonatal care in the identification of maltreatment.

Page. 2,3

Methods:
1. Design: p. 7, lines 14 – 23, the authors stated that this review was focused on young children but spanned the children's age from 0 to 18 years to prevent missing cases. Though it is reasonable to extend the age range during the literature search, it is recommended to stay focused on the targeted population, especially in the results and discussion sections, to highlight and discuss the quality and appropriateness of the existing child abuse screening tools for young children.

Response
We added a line in the "study design"-section:

For this review, we focus on young children, specifically infants- including those receiving care in neonatal settings- within pediatric healthcare.

Page. 4, section 3.

In the discussion section we added:

Our study adds updated evidence from recent publications and specifically focused on tools for neonatal clinical settings, we highlight an evidence gap for this group, despite these being a particularly vulnerable population.

Page. 10, section 6.

---

2. Search strategy:
(1) Table 1 is recommended to be placed in the appendix.
(2) Table 1d is the search strategy used for the CINAHL database, which was not mentioned in the text.

Response:
(1) Thank you for pointing this out, table 1 is placed in the appendix.
(2) Thank you for pointing this out. We acknowledge that the use of the CINAHL database was not explicitly mentioned in the main text. We have now updated the Methods section to include CINAHL as one of the databases used in the systematic search

Page. 5, section 2.

---

3. Search outcome: Figure 1 PRISMA flowchart recommends using the PRISMA 2020 flow diagram.

Response

Thank you for this observation. We confirm that the flowchart in Figure 1 is based on the PRISMA 2020 guidelines and follows the recommended structure. The PRISMA diagram is a supplementary file.

Results & discussion:
1. Results: In addition to categorizing the screening tools by their quality levels, it is recommended to identify which tools are most applicable for use with young children, in alignment with the specific focus of this study. Highlighting age-appropriate tools will enhance the practical value and relevance of the findings.

Response

Thank you for the suggestion. We have now included in the results section which tools are suitable for younger children.

Page. 9, section 6

---

2. Discussion: It is recommended to discuss the similarities and differences with the previous systematic review by Chen et al. (2022) to underline the strengths and significance of this review.

Response

Thank you for this helpful suggestion. We have now added a comparison with the review by Chen et al. (2022) in the Discussion section.

Page. 10, section 6
